# Supplementary material for: Bioinspired artificial spider silk photocatalyst for the high-efficiency capture and inactivation of bacteria aerosols
Source: Nat Commun. 2023 Apr 27;14:2412. doi: 10.1038/s41467-023-38194-1 (PMC10134728; doi:10.1038/s41467-023-38194-1)
Supplement: Supplementary file 1 — Supporting information [file 41467_2023_38194_MOESM1_ESM.pdf]

Supplementary information for "Bioinspired Artificial Spider Silk Photocatalyst for High-efficiency Capturing and Inactivation of Bacteria Aerosols"

Linghui Peng[1,2], Haiyu Wang[1,2], Guiying Li[1,2], Zhishu Liang[1,2], Weiping Zhang[1,2], Weina Zhao[1,2] and Taicheng An[1,2]✉

[1]Guangdong Key Laboratory of Environmental Catalysis and Health Risk Control, Guangdong-Hong Kong-Macao Joint Laboratory for Contaminants Exposure and Health, Institute of Environmental Health and Pollution Control, Guangdong University of Technology, Guangzhou 510006, China;

[2]Guangdong Engineering Technology Research Center for Photocatalytic Technology Integration and Equipment, Guangzhou Key Laboratory of Environmental Catalysis and Pollution Control, School of Environmental Science and Engineering, Guangdong University of Technology, Guangzhou 510006, China.

✉ E-mail: antc99@gdut.edu.cn.

Supplementary Methods

Materials. Nylon fibers with 60-80 μm in diameter were obtained from Dongguan Deyongjia Textile Co., LTD. Titanium dioxide (P25, 99.5%) and Polymethyl methacrylate (PMMA, injection grade) was purchased from Macklin Co. Ltd. N,N-Dimethylformamide (DMF, 99.8%), luminol (98%), fluorescein diacetate (FDA, 97%), ethanol (99.9%), acetone(99.9%), nutrient broth and nutrient agar were purchased from Aladdin Co. Ltd. Reactive oxygen species (ROSs) assay kit was purchased from Beyotime Co. Ltd. Trichloromethane (AR) was purchased from Guangzhou chemicals Co. Ltd. All materials were used as bought without purification.

Material characterization. Scanning electron microscopy (SEM) was performed on a Hitachi S-3400N equipped with an EDS detector. Electron paramagnetic resonance (EPR) signals of spin-trapped paramagnetic species with DMPO (5,5-Dimethyl-1-pyrroline-N-oxide), TEMP (4-Amino-2,2,6,6-tetramethylpiperidine) and TEMPO (4-Acetamido-2,2,6,6-tetramethylpiperidine 1-Oxyl free radical) were recorded with a Bruker A200 spectrometer. Water contact angle of the single fiber was carried out by the contact angle tester (Kruss DSA100) as described in followings. Atomic force microscopy (AFM) was carried out by a microscope (Dimension FastScan, Bruker), and AFM bacteria probe was observed by a laser scanning confocal microscope (OLS4100). Chemiluminescence spectra were recorded by a laser microscopic confocal Raman spectrometer (LabRAM HR Evolution). The aerodynamic diameter of the bioaerosols was investigated by an aerodynamic particle size spectrometer (TSI, APS-3321). OD600 was measured by microplate reader (Thermo Scientific Varioskan LUX).

Capture performance measurement of the ASS photocatalyst. The experimental setup

is divided into two major parts, including bioaerosol generation part and capture&inactivation part. The bioaerosol with different RHs was sprayed into the Teflon box (188 L). Four ASS photocatalysts with 5 cm long were fixed on the holder parallelly, standing in front of the bioaerosol spraying in the box. The UV lamp was placed on the back of the ASS array. The bioaerosol capture and inactivation performances of the ASS photocatalyst were characterized without using a filtration system with a strong exhaust system (negative pressure) at the end of outlet. The bioaerosol flow rates come from the bioaerosol generation system.

The diffusion dryer (PERMA PURE Nafion MD-700) with a length of 120 cm is selected for bioaerosol drying based on moisture diffusion. The bioaerosols go through the central tube and moisture is diffused into purge gas or vacuum (shell tube) due to moisture gradients, yielding bioaerosols with different RHs. Thus, the RHs of the injecting air or bioaerosol can be tuned by adjusting the air flow rate or vacuum of the purge gas in shell area. The chamber is rinsed by the bioaerosols for 30 min, thus RH in chamber can be changed by injecting bioaerosols with different RHs.

All disks and materials were sterilized in an autoclave before experiments. The bacterial cells were cultivated in nutrient broth at 37 °C for 12 h to yield a cell count of 10^3 to 10^9 CFU mL^{-1}. Then, the bacterial cells were collected by centrifugation (7104 g for 2 min) and resuspended in sterile saline solution (0.9% (w/v)). Typically, 50 mL of bacteria solution (10^9 CFU mL^{-1}) was poured into glass bottle of SKC Collison bioaerosol generator. Clean air with 3-18 L min^{-1} was injected into the generator and sprayed bioaerosols passed through the diffusion drying tube to control their RH. The assembled ASS photocatalysts (four 5-cm arrays) were placed in the bioaerosols flow for 2 min to capture airborne bacteria. Then, the ASSs

photocatalyst were immersed into 20 ml saline solutions under ultrasonic bath for 20 min, and the solution was vortexed for 1 min and then diluted for plating. The number of colonies was enumerated through visual inspection. All the experiments were repeated for three times.

Calculation of inactivation efficiency of the ASS photocatalyst. The inactivation efficiency of the ASS photocatalyst was calculated using the following Eq. (1):

$$\eta = \frac{C_0 - C_1}{C_0} \quad (1)$$

where "C_0" represents the number of living bacteria captured on the ASS photocatalyst without light irradiation, and "C_1" represents the living bacteria after light irradiation.

Water contact angle of single fiber. The water contact angle of single fiber at different RH was conducted by a water contact angle tester (Kruss DSA100) equipped with 10 pL water droplet sprayer. The advancing and receding contact angles of the ASS during bioaerosol capture process were *in situ* characterized by OCA15Pro contact angle meter.

Preparation of bacteria probe. The bacteria probe was prepared by following procedures. First, 50 mL bacteria suspension of 10^9 CFU/mL was centrifuged, and the bacteria were spread into a thin film on the glass slide. Then it was quickly dried by heated flow, and removed from the glass slide into powder. An AFM probe without the tip was dipped into epoxy and bacteria powder successively. Finally, the top of the cantilever was adhered by an aggregation of bacteria with size of around 20 μm.

Calculation of volume of water droplets. The volume of water droplets was calculated by the formula for calculating the volume of hemispheres Eq (2):

$$V = n \times \frac{1}{2} \times \frac{4}{3} \pi R^3 \quad (2)$$

where V is the volume of the total droplets of 800 μm ASS photocatalyst, n is the number

of the droplets, R is radius of the droplets.

Molecular computational details. To investigate the hydrophilicity mechanism on the joint and spindle knot, the density functional theory (DFT) calculations were performed in this work by DMol3 module in Materials Studio. The generalized gradient approximation (GGA) with the correction by the Perdew-Burke-Ernzerhof (PBE) was used as the exchange-correlation functional. The core electrons were calculated by all electron method and double numerical plus polarization (DNP) is applied as the basis set with the orbital cut off of 3.9 Å in our calculations. The convergence tolerance of energy was set to 10^{-5} Hartree (1 Hartree = 27.21 eV), and the maximum allowed force and displacement were 0.004 Hartree/Å and 0.005 Å, respectively. The smearing parameter of 0.005 Ha was taken to facilitate structure convergence. A slab model of titanium dioxide is used while a vacuum space of ~13 Å is taken to avoid the interactions between periodic slabs. The interaction energy ($\triangle E_{int}$) of water moleculars on joint and spindle knot is defined by the following Eq. (3):

$$\triangle E_{int} = E(n \cdot H_2O/sur) - E(n \cdot H_2O) - E(sur) \qquad (3)$$

where $E(n \cdot H_2O/sur)$ was the total energy of species/surface complex, E(sur) and $E(n \cdot H_2O)$ were the total energies of corresponding adsorption carrier and the sole water moleculars, respectively. The more negative value from this equation indicated the stronger interaction accordingly.

ROS measurement by Raman spectrometer. The ROSs generated on the ASS photocatalyst in air were estimated by the Laser Confocal Raman spectrometer (LabRAM HR Evolution). Luminol was employed as the indicator to reflect the generation of ROSs by chemiluminescence. The bulk ASS photocatalysts were irradiated by UV (325 nm) laser for 5

min, and then 50 µL luminol (100 µM) was dropped on the laser spot. The laser wavelength of 325 nm used in Raman equipment is closest to that used in photocatalytic inactivation experiment (365 nm). Therefore, the wavelength of 325 nm UV light for ROS generation experiment was used. Then the emission spectrum was recorded immediately, with 3 times repeated.

Surface energy gradient is given by[1,2]:

$$F=\int_{L_j}^{L_k} \gamma(\cos\theta_A-\cos\theta_R)dl \qquad (4)$$

where γ is the surface tension of water, θ_A and θ_R are the advancing angle and receding angle of the droplet on the ASS photocatalyst, respectively, and dl is the intergrating variable along the length from the joint(L_j) to the spindle knot (L_k).

Laplace pressure difference is described as[3]:

$$\triangle P= -\int_{r}^{\frac{H}{2}} \frac{2\gamma}{(R+R_0)^2} \sin\beta \, dz \qquad (5)$$

where R is the local radius, R_0 is the droplet radius, β is the angle of the spindle knot, r is radius of the fiber, H/2 is radius of spindle knot, and z is the integrating variable along the diameter of the spindle knot.

Supplementary Tables

Supplementary Table 1 The optimal preparation parameters of the ASS photocatalyst

**Fiber types**	**Diameter**	**Cross-section**	**Ratio of PMMA/DMF (by weight)**
Nylon	60 µm	Circular	10:100
**Ratio of TiO_2/PMMA solution (by volume)**	**Drawing speed**	**Drawing angle**	
1:100	30 cm s^{-1}	0°	

Supplementary Table 2 The preparation parameters of the ASS photocatalyst with different morphologies

Sample	H (μm)	L (μm)	β (°)	Drawing speed (cm s^{-1})	PMMA concentration (g/ 100 g solution)
1	90	150	32	30	5
2	90	250	33	50	5
3	120	200	42	30	10
4	120	300	46	50	10

Supplementary Table 3 The calculated volume of droplets captured by the ASS photocatalyst with same length

Sample	Number of droplets	Radius of droplets (μm)	Volume of droplets (μL)
1	4	70	0.25
2	3.5	70	0.22
3	2.5	133	1.24
4	2	133	0.99

Supplementary Figures

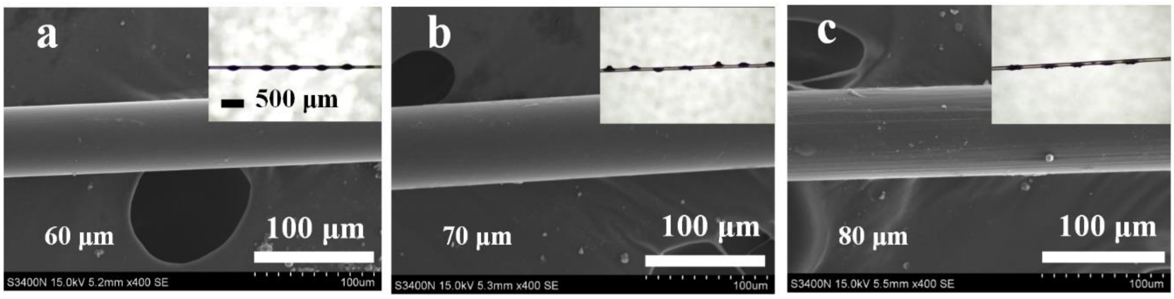

Supplementary Fig. 1 Optical and SEM images of the ASS photocatalyst with different diameters: **(a)** 60, **(b)** 70 and **(c)** 80 µm (The independent experiment has been repeated for three times, and similar results were obtained).

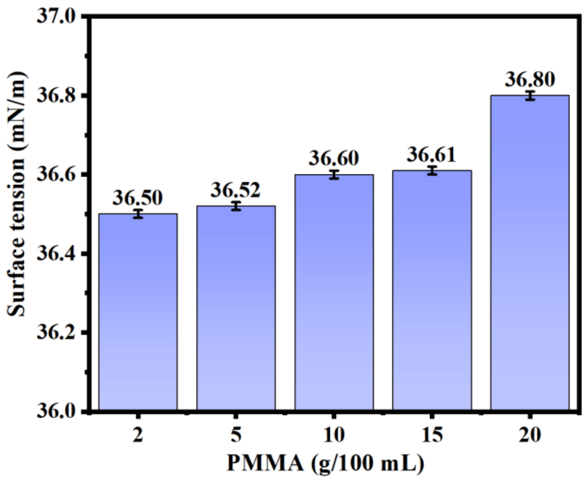

Supplementary Fig. 2. Surface tensions of the PMMA/TiO_2 mixture with different concentration of PMMA. The error bars are calculated via repeating the measurements for three times.

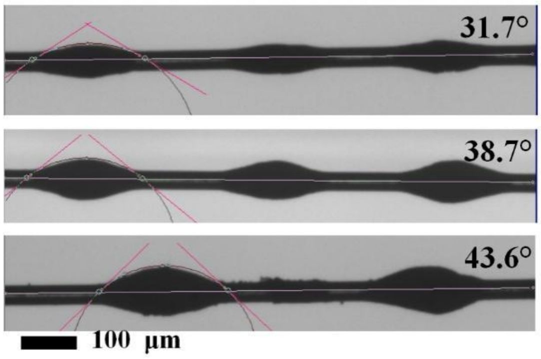

Supplementary Fig. 3. Angle (β) of the spindle knot and length (L) of joints with different concentration of PMMA (The independent experiment has been repeated for three times, and similar results were obtained).

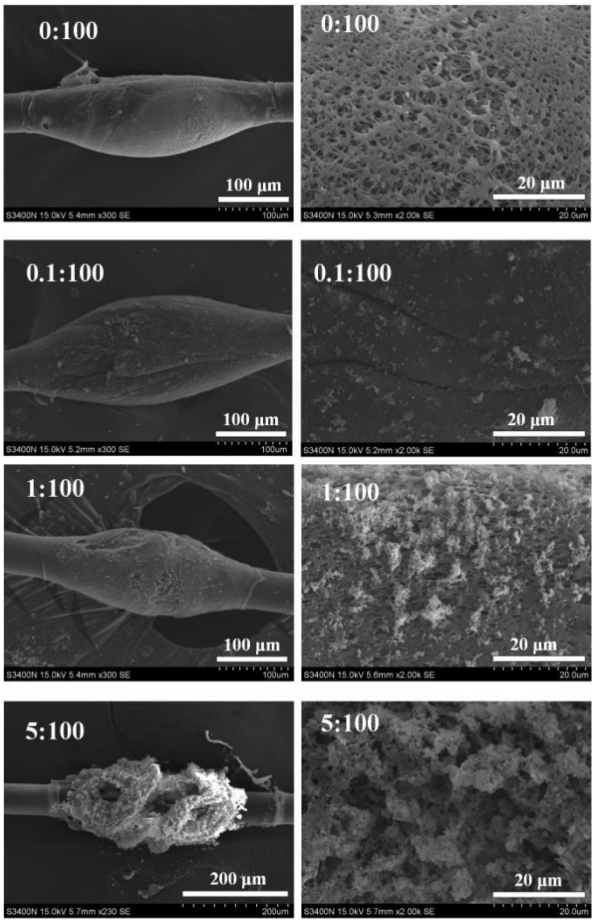

Supplementary Fig. 4. Surface morphology of the ASS with different TiO_2 concentrations (The independent experiment has been repeated for three times, and similar results were obtained).

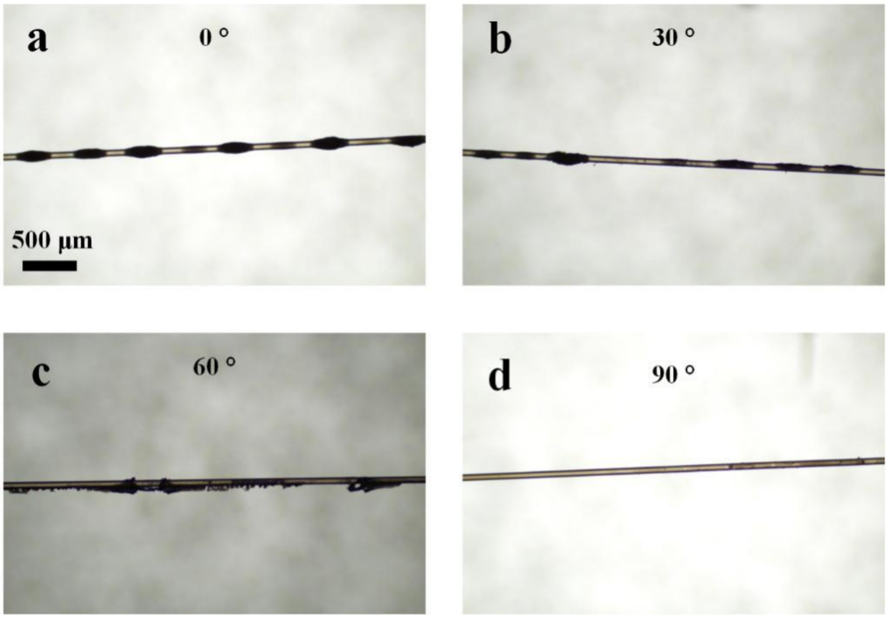

Supplementary Fig. 5. Optical images of the ASS photocatalyst fabricated with different drawing angles: **(a)** 0°, **(b)** 30°, **(c)** 60°, and **(d)** 90° (The independent experiment has been repeated for three times, and similar results were obtained).

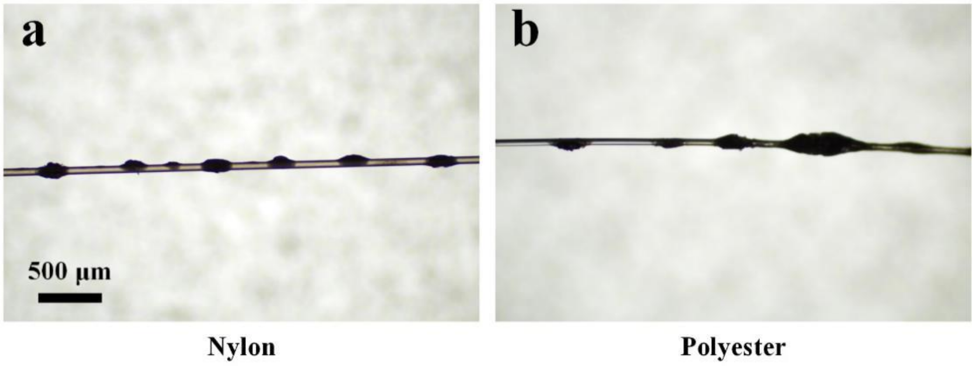

Supplementary Fig. 6. Optical images of the ASS photocatalyst fabricated by **(a)** nylon and **(b)** polyester(The independent experiment has been repeated for three times, and similar results were obtained).

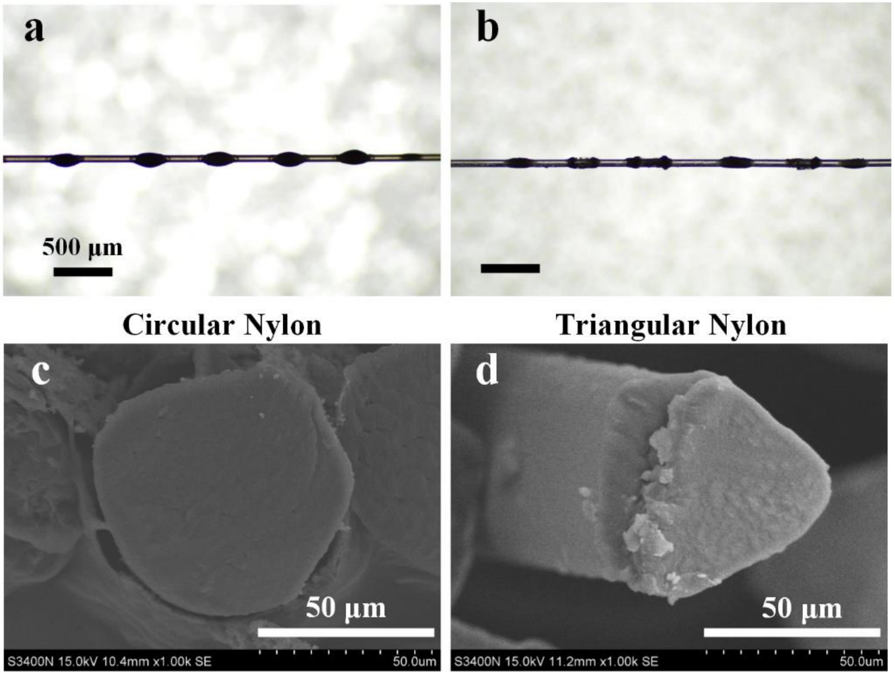

Supplementary Fig. 7. Optical and SEM images of the ASS photocatalyst with different cross-sections: **(a), (c)** Circular nylon and **(b), (d)** Triangular nylon (The independent experiment has been repeated for three times, and similar results were obtained).

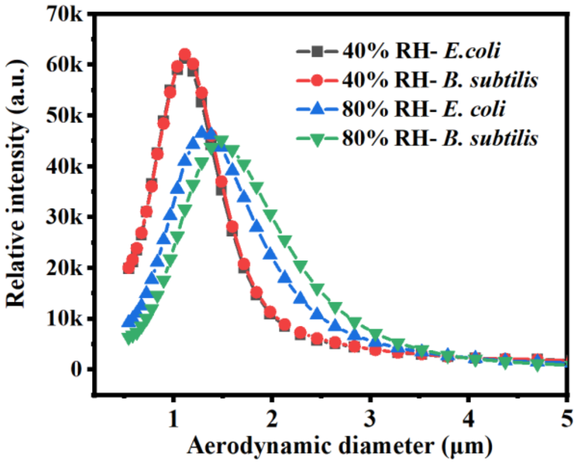

Supplementary Fig. 8. Aerodynamic diameters of *E. coli* and *B. subtilis* bioaerosols at different relative humidity.

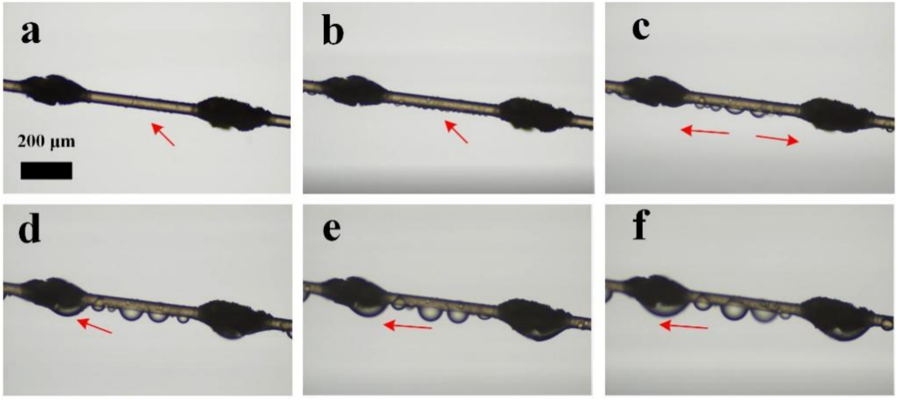

Supplementary Fig. 9. Optical images of the bioaerosols capture procedures of the ASS photocatalyst: **(a)** beginning, **(b)** micro-droplet growing, **(c)** moving, **(d)** condensing, **(e)** collecting, and **(f)** big droplet growing. (The independent experiment has been repeated for three times, and similar results were obtained).

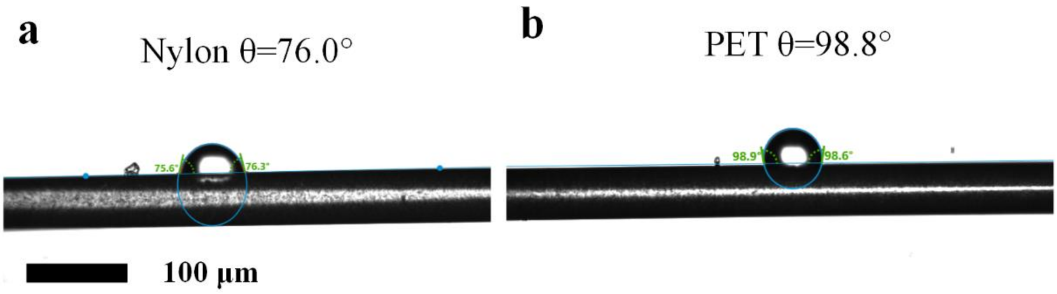

Supplementary Fig. 10. Water contact angles of the single **(a)** nylon and **(b)** polyester fiber (The independent experiment has been repeated for three times, and similar results were obtained).

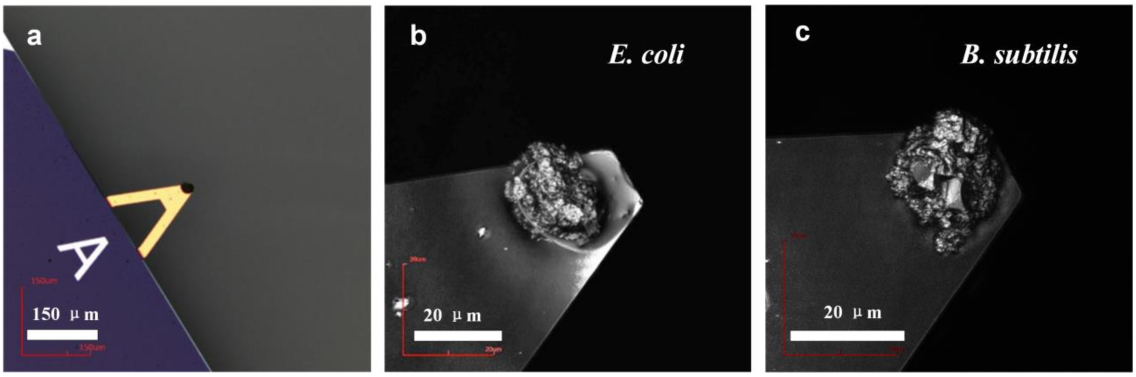

Supplementary Fig. 11. Optical images of bacterial probes for AMF characterization. *E. coli* bacterial probe at **(a)** low and **(b)** high magnification, and **(c)** *B. subtilis* bacterial probe.

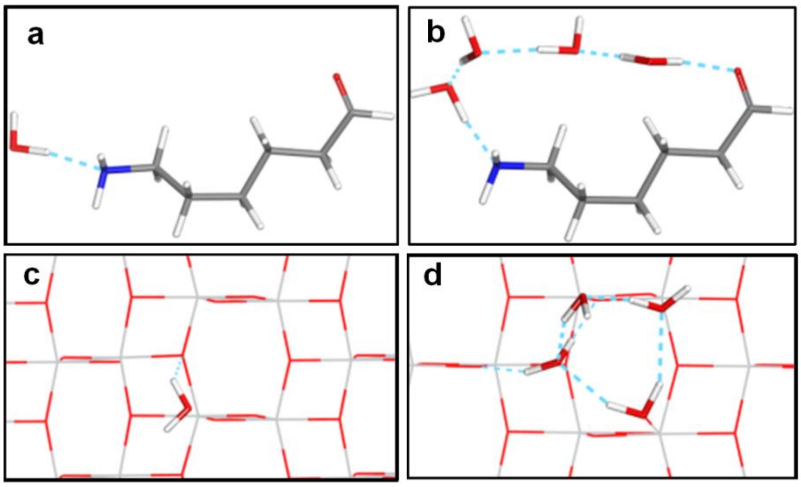

Supplementary Fig. 12. Theoretical model of the local hydrogen bonds: at the low **(a)** and high **(b)** RH situations on the joint, at the low **(c)** and high **(d)** RH situations on the spindle knot. The dark brown, light brown, blue, white and red stick balls are C, Ti, N, H and O atoms, respectively. The dotted green line indicates the hydrogen bonds.

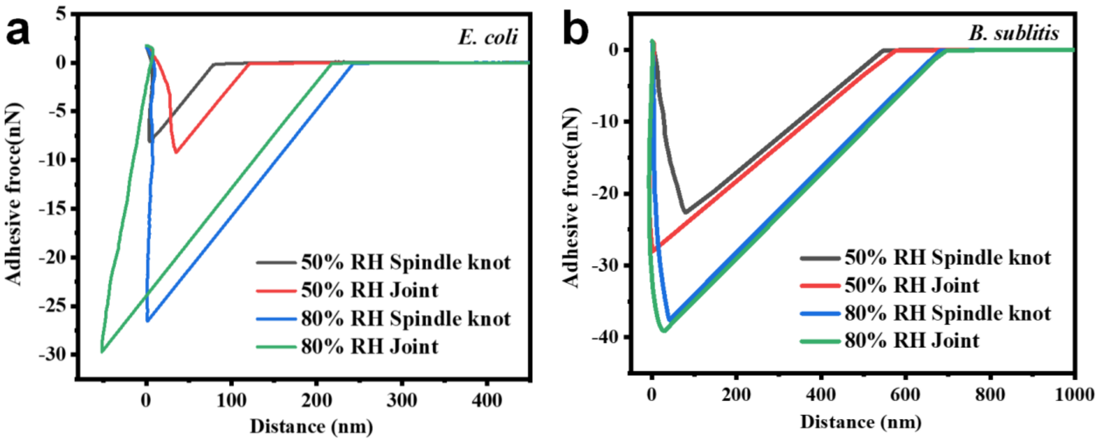

Supplementary Fig. 13. Representative adhesive forces between the **(a)** *E. coli* and **(b)** *B. subtilis* bacterial probe and the ASS photocatalyst at different RH.

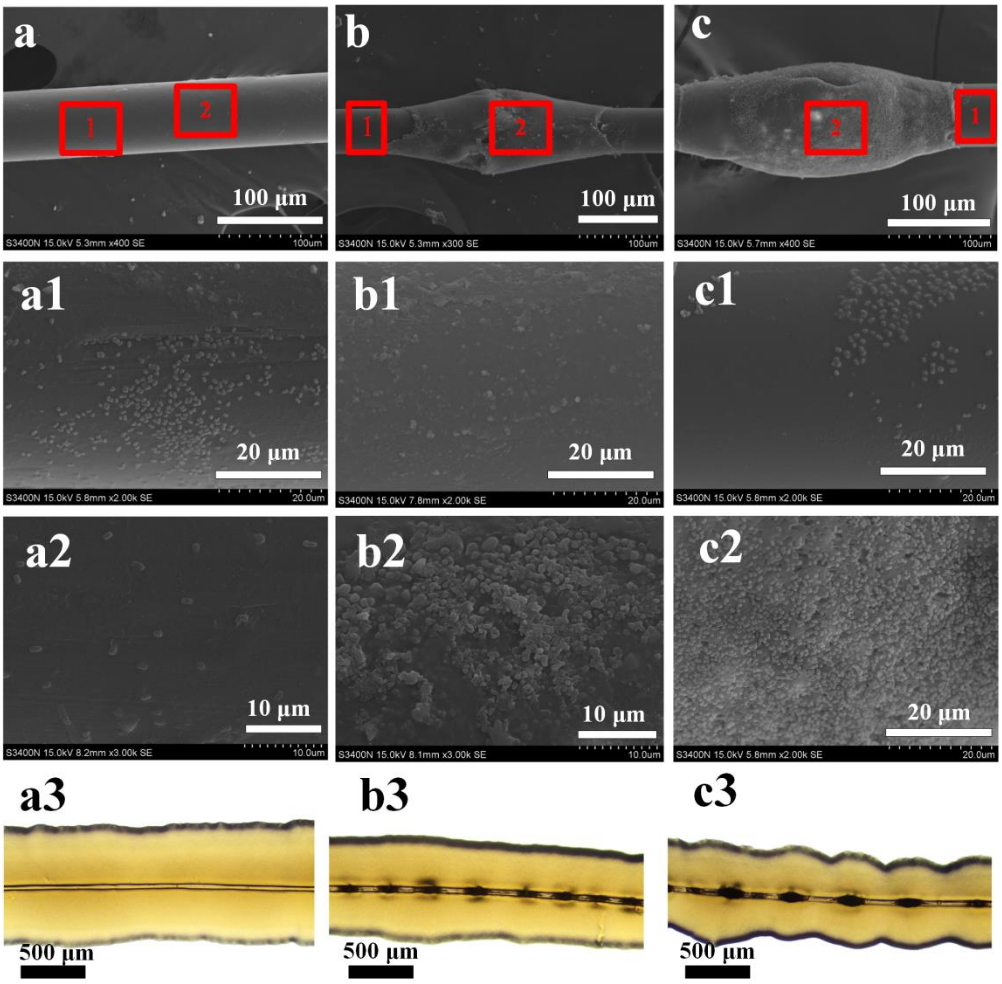

Supplementary Fig. 14. Optical and SEM images of bacteria distribution on the pure nylon and the ASS photocatalyst: SEM images of **(a)** the pure nylon, and **(a1)** & **(a2)** bacteria on the nylon fiber. **(a3)** Optical image of the pure nylon cultivated on nutrient agar. SEM images of **(b)** the ASS photocatalyst with small spindle knots, and **(b1)** & **(b2)** bacteria on the ASS with small spindle knots. **(b3)** Optical image of the ASS cultivated on nutrient agar. SEM images of **(c)** the ASS photocatalyst with big spindle knots, and **(c1)** & **(c2)** bacteria on the ASS with big spindle knots. **(c3)** Optical image of the ASS cultivated on nutrient agar. (The independent experiment has been repeated for three times, and similar results were obtained).

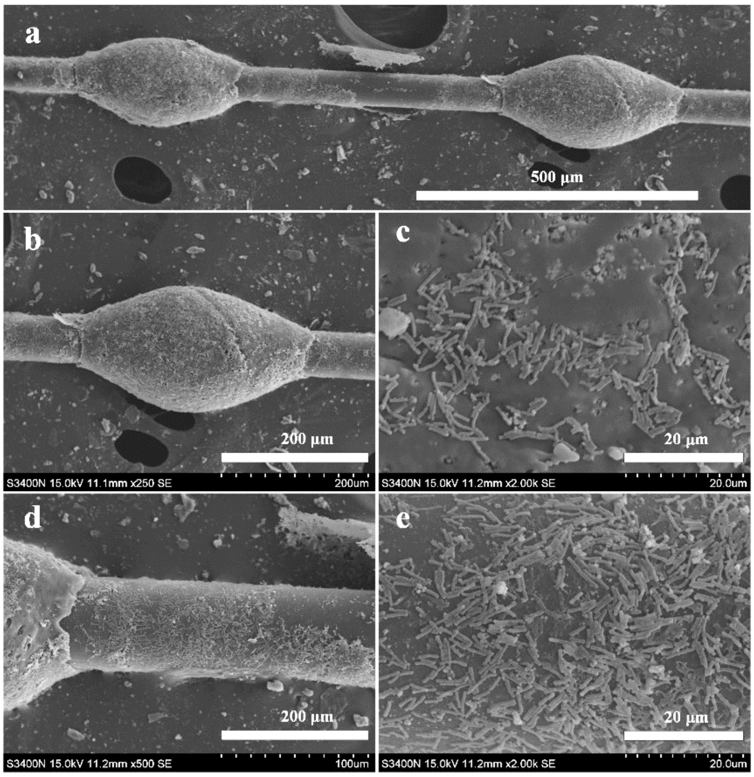

Supplementary Fig. 15. SEM images of bacteria distribution on the ASS photocatalyst with long joints: **(a)** overall view of the ASS photocatalyst, **(b)** spindle knot, **(c)** bacteria on the spindle knot at high magnification, **(d)** the joint, and **(e)** bacteria on the joint at high magnification (The independent experiment has been repeated for three times, and similar results were obtained).

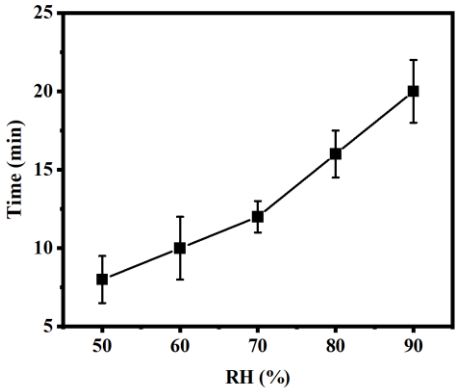

Supplementary Fig. 16. Time of droplet evaporation at different RHs (15 °C). The error bars are calculated via repeating the measurements for three times (n=3), and data are presented as mean values +/- SD.

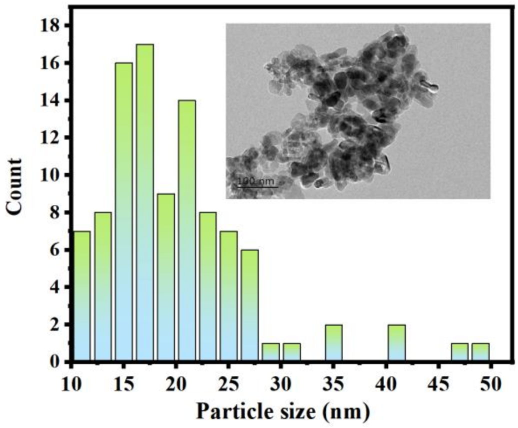

Supplementary Fig. 17. Particle size distribution of P25 TiO_2 nanoparticles.

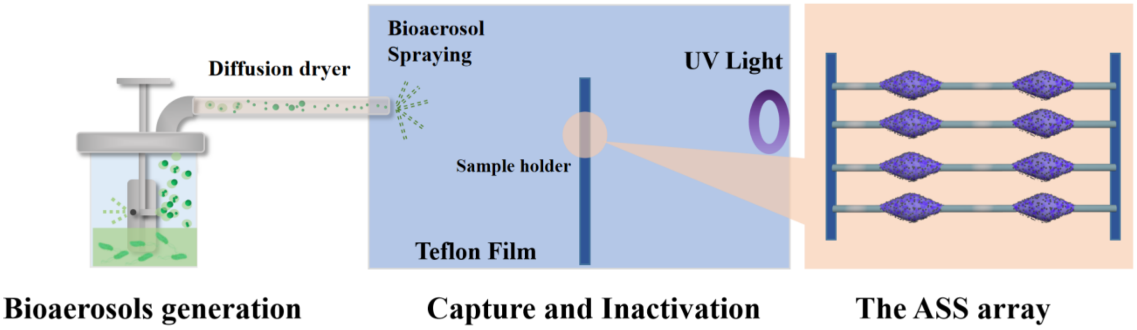

Supplementary Fig. 18. Illustration of experimental setup.

Supplementary Discussion

To provide insights into the adhesive force under the interested conditions of low RH or high RH, we have first calculated the adsorption geometrical structure of one water molecule and then found the most favorable adsorption structure of H_2O on the joint (Supplementary Fig. 12 (a))/spindle knot (Supplementary Fig. 12 (c)) from several possible configurations. It clearly shows that water adsorbs on the joint via one H atom forming a hydrogen bond with one N atom of the joint, while there is also a hydrogen bond for water with one O atom of the spindle

knot. In accordance with the experiment results, the interaction energy of water molecular on the joint is obtained as -0.93 eV with the distance H---NH of ~1.9 Å under the low RH, higher than that of water molecular on the spindle knot of -0.80 eV with the distance H---OH of ~ 2.3 Å. At high RH conditions, we found that the other water molecules near to the adsorbed water interact with the adsorbed water via hydrogen bonding, forming a liquid bridge on the joint (Supplementary Fig. 12 (b)). Similarly, a weakly-bound water layer is also found on the spindle knot (Supplementary Fig. 12 (d)), but with a less hydrogen bonds and far distance above the surface. In consist with the structural change, we found that the interaction between water molecules on both the joint and spindle knot are enhanced to -5.32 and -4.09 eV, respectively, which is contributed to the more hydrogen bonds. From the above, it clearly indicates that at both low RH and high RH, the adhesive force on the joint was stronger that on the spindle knot. At high RH, the adhesive force was significantly stronger than that at low RH due to the more hydrogen bonds responsible for capillary force.

Supplementary References

1. Zheng, Y. et al. Directional water collection on wetted spider silk. *Nature*, **463**(7281),640-643 (2010).
2. Daniel, S., Chaudhury, M.K.& Chen, J.C. Fast drop movements resulting from the phase change on a gradient surface. *Science*, **291**,633-636(2001).
3. Lorenceau, E., & Quéré, D. Drops on a conical wire. *J. Fluid Mech.* **510**, 29-45(2004).
