# Supplementary material for: Bioinspired artificial spider silk photocatalyst for the high-efficiency capture and inactivation of bacteria aerosols
Source: Nat Commun. 2023 Apr 27;14:2412. doi: 10.1038/s41467-023-38194-1 (PMC10134728; doi:10.1038/s41467-023-38194-1)
Supplement: Supplementary file 3 — Description of Additional Supplementary Files [file 41467_2023_38194_MOESM3_ESM.pdf]

Description of Additional Supplementary Files

File Name: Supplementary Movie 1
Description: Movie of bioaerosols captured by the ASS photocatalyst.
