# Supplementary material for: Bioinspired artificial spider silk photocatalyst for the high-efficiency capture and inactivation of bacteria aerosols
Source: Nat Commun. 2023 Apr 27;14:2412. doi: 10.1038/s41467-023-38194-1 (PMC10134728; doi:10.1038/s41467-023-38194-1)
Supplement: Supplementary file 5 — Reporting Summary [file 41467_2023_38194_MOESM5_ESM.pdf]

nature portfolio

Corresponding author(s): Taicheng An

Last updated by author(s): Apr 6, 2023

Reporting Summary

Nature Portfolio wishes to improve the reproducibility of the work that we publish. This form provides structure for consistency and transparency in reporting. For further information on Nature Portfolio policies, see our Editorial Policies and the Editorial Policy Checklist.

Statistics

For all statistical analyses, confirm that the following items are present in the figure legend, table legend, main text, or Methods section.

n/a	Confirmed	
☐	☒	The exact sample size (*n*) for each experimental group/condition, given as a discrete number and unit of measurement
☐	☒	A statement on whether measurements were taken from distinct samples or whether the same sample was measured repeatedly
☒	☐	The statistical test(s) used AND whether they are one- or two-sided<br>*Only common tests should be described solely by name; describe more complex techniques in the Methods section.*
☐	☒	A description of all covariates tested
☐	☒	A description of any assumptions or corrections, such as tests of normality and adjustment for multiple comparisons
☐	☒	A full description of the statistical parameters including central tendency (e.g. means) or other basic estimates (e.g. regression coefficient) AND variation (e.g. standard deviation) or associated estimates of uncertainty (e.g. confidence intervals)
☒	☐	For null hypothesis testing, the test statistic (e.g. *F*, *t*, *r*) with confidence intervals, effect sizes, degrees of freedom and *P* value noted<br>*Give P values as exact values whenever suitable.*
☒	☐	For Bayesian analysis, information on the choice of priors and Markov chain Monte Carlo settings
☒	☐	For hierarchical and complex designs, identification of the appropriate level for tests and full reporting of outcomes
☒	☐	Estimates of effect sizes (e.g. Cohen's *d*, Pearson's *r*), indicating how they were calculated

Our web collection on statistics for biologists contains articles on many of the points above.

Software and code

Policy information about availability of computer code

Data collection	DMol3 module in Materials Studio
Data analysis	ImageJ V1.50i

For manuscripts utilizing custom algorithms or software that are central to the research but not yet described in published literature, software must be made available to editors and reviewers. We strongly encourage code deposition in a community repository (e.g. GitHub). See the Nature Portfolio guidelines for submitting code & software for further information.

Data

Policy information about availability of data

All manuscripts must include a data availability statement. This statement should provide the following information, where applicable:

- Accession codes, unique identifiers, or web links for publicly available datasets
- A description of any restrictions on data availability
- For clinical datasets or third party data, please ensure that the statement adheres to our policy

The source data generated in this study are provided with this paper. The additional data are provided in the Supplementary Information. All the data sets generated and analysed during the current study are available from the corresponding authors on request.

Human research participants

Policy information about studies involving human research participants and Sex and Gender in Research.

Reporting on sex and gender	N/A
Population characteristics	N/A
Recruitment	N/A
Ethics oversight	N/A

Note that full information on the approval of the study protocol must also be provided in the manuscript.

Field-specific reporting

Please select the one below that is the best fit for your research. If you are not sure, read the appropriate sections before making your selection.

☐ Life sciences ☐ Behavioural & social sciences ☒ Ecological, evolutionary & environmental sciences

For a reference copy of the document with all sections, see nature.com/documents/nr-reporting-summary-flat.pdf

Ecological, evolutionary & environmental sciences study design

All studies must disclose on these points even when the disclosure is negative.

Study description	We performed a laboratory study of the airborne bacteria capture and inactivation efficiency of the artificial spider silk photocatalyst by generating bacteria aerosols from the stock bacterial suspension.
Research sample	Two cultured laboratory strains of E. coli and B. subtilis bacteria were used.
Sampling strategy	Separate cultures were prepared for each individual experiment on different days.
Data collection	For each bacteria cultivation experiment, bacteria were sampled in random order and plated in random order. The counting data were collected by Linghui Peng. The optical images and movie of bacteria captured by the photocatalyst were collected by Linghui Peng using a microscope with a camera.
Timing and spatial scale	For each individual experiment, all data was collected on the same day with an individual cell culture prepared on the day of the experiment. The eluted bacteria were mixed uniformly after being captured by the photocatalyst and were plate counting within 2 h to ensure bacteria viability. Furthermore, the order of plating bacteria was randomized.
Data exclusions	No data exclusions.
Reproducibility	All experiments were performed with at least three technical replicates to ensure reproducibility across experiments. The experiments can be reproduced by keeping the samples in the same conditions.
Randomization	The bacterial culture was randomly chosen from the plate for generation of bioaerosols.
Blinding	Blinding was not relevant as all processing methods were done through available software with consistent parameters utilized across all treatment groups.

Did the study involve field work? ☐ Yes ☒ No

Reporting for specific materials, systems and methods

We require information from authors about some types of materials, experimental systems and methods used in many studies. Here, indicate whether each material, system or method listed is relevant to your study. If you are not sure if a list item applies to your research, read the appropriate section before selecting a response.

Materials & experimental systems

n/a	Involved in the study
☒	☐ Antibodies
☒	☐ Eukaryotic cell lines
☒	☐ Palaeontology and archaeology
☒	☐ Animals and other organisms
☒	☐ Clinical data
☒	☐ Dual use research of concern

Methods

n/a	Involved in the study
☒	☐ ChIP-seq
☒	☐ Flow cytometry
☒	☐ MRI-based neuroimaging
